# Tobacco control and prevention efforts in Ethiopia pre- and post-ratification of WHO FCTC: Current challenges and future directions

**DOI:** 10.18332/tid/102286

**Published:** 2019-02-22

**Authors:** Daniel Asfaw Erku, Eyasu Teshome Tesfaye

**Affiliations:** 1 School of Pharmacy, University of Gondar, Gondar, Ethiopia; 2 College of Law, Dire Dawa University, Dire Dawa, Ethiopia

**Keywords:** tobacco control, Ethiopia, tobacco industry, WHO FCTC

## Abstract

**INTRODUCTION:**

Being the second most populous African country, Ethiopia represents a huge opportunity for the tobacco industry to recruit new smokers. Ethiopia signed the convention to ratify WHO Framework Convention on Tobacco Control (FCTC) in 2004 and ratified in 2014. We reviewed Ethiopia’s tobacco control legislative history pre- and post-ratification of the WHO FCTC and evaluated the level of compliance of the National Tobacco Control Directive (NTCD) with the WHO FCTC.

**METHODS:**

We reviewed Ethiopia’s tobacco legislative history, the NCTD, the National Tobacco Control Strategic Plan, and tobacco control related media stories from 2009 to 2018. The level of compliance of NTCD with WHO FCTC was compared and qualitatively analysed.

**RESULTS:**

NTCD 2015 is Ethiopia’s first comprehensive tobacco control legislation, which for the most part is WHO FCTC compliant. The legislation prohibits, among other things, sale of flavoured tobacco products including menthol, sale of tobacco products to a person under the age of 18 years and bans all forms of tobacco advertising, promotion, and sponsorship. Yet, the current legislation allows smoking designated rooms in some prohibited places. Although a multi-sectoral National Tobacco Control Committee and a Strategic Plan were developed as per Article 5 of WHO FCTC, activities pertaining to the protection of such tobacco control policies from vested interests of the tobacco industry (WHO FCTC Article 5.3) are not addressed in NTCD 2015.

**CONCLUSIONS:**

Major gaps in the NTCD 2015 such as allowing smoking designated rooms should be addressed in order to stop the tobacco industry from using such loopholes to interfere with national tobacco control policies and/or maintain its tobacco market. Moreover, the tobacco control policies and efforts should be institutionalized across various sectors in order to ensure implementation of the NTCD.

## INTRODUCTION

Tobacco use is one of the most dreadful, yet avoidable causes of death and disease^1^. From 1.1 billion smokers worldwide, nearly 80% of them currently live in lowand middle-income countries (LMICs)^2,3^. Although prevalence of smoking in Africa is amongst one of the lowest in the world^4^, the rapidly growing economies of most African countries, along with the increasing interest of global tobacco companies to gain a share in the African tobacco market, have the potential to contribute to a surge in the tobacco epidemic of the region. Among the LMICs, Sub-Saharan African countries are regions that are currently facing a substantial surge in tobacco use^5^, and Ethiopia is no exception.

Data regarding trends in tobacco use are limited in Ethiopia. The Global Adult Tobacco Survey (GATS)-Ethiopia, which was published in 2016, is the first comprehensive and nationally representative household survey of people of age 15 years or older. According to GATS, 5% (3.4 million) of adults in Ethiopia use tobacco products (ranging from 1% in Amhara region to 18% in Somali region) and 6.5 million people were exposed to secondhand smoke^6^. Slightly lower smoking rates were also reported in the WHO non-communicable diseases (NCDs) STEPS survey (4.2%) and Ethiopian Demographic and Health Survey (4%)^7,8^. A disproportionately high prevalence of tobacco use was also reported in some regions of Ethiopia such as Afar (15.5%) and Gambella (11.2%). Due to the country’s increasing rate of smoking and demand, National Tobacco Enterprise (NTE), Ethiopia’s premier tobacco company, expanded its market from 4 billion cigarettes to 6 billion annually^9^. Being the second most populous African country after Nigeria, Ethiopia also represents a huge opportunity for international tobacco companies to invest and recruit new smokers. In 2016, Japan Tobacco International (JTI) become a major stakeholder in the country’s tobacco market by acquiring nearly 40% of NTE for $434 million USD^10^.

Ethiopia is one of the many countries that signed the convention to ratify WHO FCTC early in 2004. However, the monopoly of the country’s tobacco industry by the state-owned NTE, which for a long time has been justified as ‘a source of tax revenue and employment opportunity’^11^ and the lack of political will inside the government have resulted in major historical setbacks to ratifying and implementing WHO FCTC. In early 2014, Ethiopia made a significant step toward tobacco control when the House of Peoples’ Representatives passed a Bill, the WHO FCTC (Proclamation No 822/2014), and gave the mandate for implementing the WHO FCTC to the Ethiopian Food, Medicine and Healthcare Administration and Control Authority (FMHACA). Subsequently, FMHACA issued the Tobacco Control Directive on 21 March 2015 in accordance with Article 4 of the WHO FCTC^12^.

The aim of this study is to review Ethiopia’s tobacco control and prevention efforts pre- and post-ratification of WHO FCTC and to evaluate the level of compliance of the National Tobacco Directive (NTCD) 2015 with the WHO FCTC. The current challenges and future directions regarding implementing the National Tobacco Directive and other tobacco control activities are also discussed.

## METHODS

Tobacco control related legislative documents were obtained from government official websites such as the Ministry of Health (http://www.moh.gov.et/) and the Ethiopian Food, Medicine and Healthcare Administration and Control Authority (http://www.fmhaca.gov.et/). Some tobacco control laws were also retrieved from the Campaign for Tobacco-Free Kids (CTFK) Website (https://www.tobaccocontrollaws.org/).

We searched two online databases (PubMed and Google Scholar) and used the Google search engine for tobacco control related media stories (Ethiopian and African newspaper Web sites) using the key words: ‘Ethiopia’, ‘Tobacco Control’, ‘FCTC’, ‘WHO FCTC implementation’, ‘Tobacco Industry’, and ‘National Tobacco Enterprise’. The search terms were customised for each database to retrieve published articles and policy documents. No date restrictions were applied.

Data regarding Ethiopia’s MPOWER implementation were retrieved from the WHO Report on the Global Tobacco Epidemic (2017 edition)^13^. Key sources of information and stakeholders were also contacted to further locate unpublished policy documents.

While the historical aspects of Ethiopia’s effort to regulate tobacco products were presented narratively, the content and level of compliance of the National Tobacco Directive (NTCD) 2015 with the WHO FCTC were qualitatively analysed. Data from policy documents were first extracted independently by both authors and disagreements were resolved through discussion and consensus. Level of compliance across all WHO FCTC Articles were presented separately and potential discrepancies and policy gaps were further discussed. Ethical approval to conduct this study was granted from the ethical review committee of the School of Pharmacy, University of Gondar, Ethiopia.

## RESULTS

### Historical accounts

The timeline and overview of tobacco control legislations in Ethiopia are summarised in Table 1. Earlier proclamations and tobacco control efforts by Ethiopian governments aimed at monopolising the tobacco industry rather than protecting the public from the dangers of tobacco. In 1942, the *Tobacco Regie* (Proclamation No. 30/1942) was established as a State Monopoly to prepare, manufacture, import, distribute and export tobacco products. This proclamation (known as the *Regie*) was later repealed in 1980 by Proclamation No. 197/1980 with the establishment of the National Tobacco and Matches Corporation but the core rationale remained the same, to give the State the power to exclusively grow and process tobacco in Ethiopia. In 1999, the monopoly right of the National Tobacco Enterprise was transferred to the National Tobacco Enterprise (Ethiopia) shares company under the proclamation No. 181/1999. Once again, these proclamations were not aimed at protecting the public from the health risks of tobacco or to acknowledge the individual and population-level dangers of tobacco use. The National Tobacco Enterprise (NTE) Share Company was established with the purpose of monopolising and regulating the production and distribution Ethiopia’s tobacco demand. This monopoly of the country’s tobacco industry by NTE has been justified as a source of tax revenue and employment opportunity^11^. In summary, all these prior attempts to regulate tobacco products aimed almost exclusively at sustaining the financial gain from tobacco products, and in no way were made for the benefit of the public.

**Table 1:** Timeline and overview of tobacco control legislations in Ethiopia

*Year/Proclamation*	*Policies and main contents*
1942 Proclamation 30/1942	Tobacco Regie Proclamation was enacted to give the State Monopoly to prepare, manufacture, import, distribute and export tobacco products
1980 Proclamation 197/1980	The National Tobacco and Matches Corporation was established and mandated to exclusively grow and process tobacco in Ethiopia
1999 Proclamation 181/1999	The monopoly right of the National Tobacco Enterprise was transferred to the National Tobacco Enterprise (Ethiopia) share Company
2004	Ethiopia signed the convention to ratify WHO FCTC
2009 Proclamation 661/2009	FMHACA was given the authority to regulate the content, manufacture, import, export, distribution, sales, use, packaging and labelling, and disposal of tobacco products
2012 Proclamation 759/2012	Advertisement and promotion of cigarette or other tobacco products was also prohibited
2013 Regulation 299/2013	FMHACA was given a full mandate to place licensing requirements on sellers, prohibit the sale of tobacco products to minors, require text and/or picture warnings on packs, and restrict smoking in certain indoor places
2014 Proclamation 822/2014	The House of Peoples’ Representatives passed a Bill for ratifying the FCTC, and FMHACA was authorised to take all necessary steps for its implementation
2015 NTCD 28/2015	FMHACA issued the National Tobacco Control Directive No 28/2015 (NTCD) in accordance with Article 4 of the FCTC
2017	NTCCC was established at the federal level and prepared the first draft of National Tobacco Control Strategic Plan (2017–2020)
2018	The Ministry of Health proposed the *Food and Medicine Administration Proclamation* that comprises a variety of legislations regarding the sale and use of alcohol, food, narcotic drugs and tobacco products

### Tobacco control Bills and legal frameworks (2009–2015)

For the first time in Ethiopia’s tobacco control history, the mandate to oversee tobacco production and distribution was taken from the tobacco producers (i.e. NTE) in 2009. The Proclamation 661/2009^14^ gave FMHACA the authority to regulate tobacco products and made responsible for the regulation of ‘the content, manufacture, import, export, distribution, sales, use, packaging and labeling, and disposal of tobacco products’. However, FMHACA’s earlier tobacco control activities were largely confined to regulating the production and distribution of tobacco products, and neglected the public health implications of tobacco use. It is only after 2013 that FMHACA had a full mandate, issued by the Council of Ministers under Regulation 299/2013, to place a licensing requirement on sellers, prohibit the sale of tobacco products to minors, require 30% tax and/or picture warnings on packs, and restrict smoking in certain indoor places^15^.

### National Tobacco Control Directive 2015

Ethiopia made a significant step toward tobacco control when the House of Peoples’ Representatives passed a Bill in early 2014 for ratifying the WHO FCTC (Proclamation No 822/2014). Once again, FMHACA was authorised to take all necessary steps for the implementation of the WHO FCTC. On 21 March 2015, the National Tobacco Control Directive No 28/2015 (NTCD) was then issued by FMHACA in accordance with Article 4 of the WHO FCTC^12^.

### General obligations

According to Article 5 of WHO FCTC, each party is required to develop, implement and review multi-sectoral national tobacco control strategies and programs that will reduce tobacco use, addiction to nicotine, and exposure to secondhand smoke^16^. Even though the NTCD 2015 document did not include details regarding such tobacco control strategies and committees, FMHACA established a multi-sectoral National Tobacco Control Coordinating Committee (NTCCC) at the federal level just after the release of NTCD 2015. The committee included stakeholders from a variety of organizations including Federal Ministry of Health, Ethiopian Public Health Institute, Ethiopian Public Health Association, Ethiopian Revenue and Customs Authority, Ministry of Labor and Social Affairs, Ministry of Youth and Sports, tobacco control advocacy groups and professional societies in order to institutionalize and mainstream tobacco control efforts in various sectors and institutions. The committee then released a draft document on the first National Tobacco Control Strategic Plan (2017–2020) on 31 October 2017. The strategic plan aims ‘to guide program managers, public health professionals, partners and stakeholders in their efforts to plan and implement tobacco control strategies to attain the objective of the WHO FCTC’ and has its own implementation, monitoring and evaluation framework. It has 9 main objectives and 23 specific objectives that mainly aim at reducing demand, discouraging tobacco use, and reducing supply of tobacco products (Table 2).

**Table 2:** Strategic objectives and strategies outlined in the National Tobacco Control Strategic Plan (2017–2020)

*Strategic objectives*	*Specific strategies*
1. To protect people and environment from tobacco exposure	1.1 Sensitize, familiarize and advocate for enforcement of laws
	1.2 Enact and enforce smoke-free public places and environment
	1.3 Protect environment and persons from hazards of tobacco cultivation & manufacturing
2. To reduce the number of people using tobacco	2.1 Promoting creation of supportive environment
	2.2 Increasing access to cessation services
3. To warn about the dangers of tobacco	3.1 Ensuring effective pack warning labels
	3.2 Implementing counter-advertising campaigns
	3.3 Regulation and disclosure of the content of tobacco products
4. To enforce bans on tobacco advertising, promotion & sponsorship	4.1 Enact and enforce effective legislation banning all forms of direct tobacco marketing advertisement
	4.2 Enact and enforce effective legislation banning indirect tobacco advertising, promotion and sponsorship
5. To discourage demand for tobacco through price and tax measures	5.1 Increase and adjust periodically tax rates for tobacco products
6. To reduce supply of tobacco products	6.1 Curb illicit trade in tobacco products
	6.2 Ban sale of duty-free tobacco products
	6.3 Ban sale of tobacco to minors
	6.4 Design and support alternative livelihoods to tobacco
7. To promote partnerships & coordination for sustained tobacco control	7.1 Strengthen a national and establish a regional coordination mechanism
	7.2 Mainstreaming of tobacco control and strengthening networking
8. Integrated communication	8.1 Promoting engagement of communities in tobacco control initiatives
	8.2 Children and Adolescent tobacco awareness raising programs
	8.3 Advocating for increased political commitment and mobilization
	8.4 Development of integrated tobacco control database and networking
9. To strengthen generation of evidence through survey and research	9.1 Establishing and strengthening national tobacco surveillance system
	9.2 Dissemination and use of findings of generated evidence

Moreover, the committee proposed to use nearly $9 million USD from government and development partners as well as tobacco tax to implement the strategic plan. While the establishment of NTCCC with members from a variety of institutions and proposed plans to raise financial resources are strengths in implementing Article 5 of WHO FCTC, efforts and proposed activities pertaining to the protection of tobacco control policies from the vested interests of the tobacco industry are not addressed in NTCD 2015.

### Price and non-price measures to reduce tobacco demand

Articles 6 and 7 of WHO FCTC require parties to implement price and tax policies, and non-price measures on tobacco products, that aim at reducing tobacco consumption. The NTCD did not include details on this matter. However, the NTCCC clearly stipulates, in the strategic plan 2017–2020, that approaches be taken to discourage demand for tobacco through price and tax measures (strategic objective 5) and reduce supply of tobacco products (strategic objective 6) by eliminating illicit trade in tobacco products, including smuggling, illicit manufacturing and counterfeiting. Currently, Ethiopia restricts importations of tobacco products by international adult passengers to 400 cigarettes or 250 grams of tobacco, without incurring customs duty.

### Protection from exposure to tobacco smoke

Article 8 of WHO FCTC requires parties to adopt and implement legislation that will protect people from exposure to tobacco smoke in indoor workplaces, conveyances, and other public places. The NTCD prohibits smoking in health and education institutions, restaurants, bars, cafes, prisons, government offices, reception areas, shopping malls, cinemas or other places of entertainment, sports places, and conveyances. Despite the requirement of 100% smoke-free places, it allows smoking if the owner of the business has smoking designated rooms and areas equipped with separate ventilation. Exceptions to this, according to NCTD, are facilities that provide services primarily to children or youth under the age of 18 years, government offices, rooms of education, and healthcare services.

### Regulation of tobacco products content and disclosure

Article 9 of FCTC requires parties to measure and regulate the contents and emissions from tobacco products. Article 10 also requires parties to implement legislation requiring tobacco producers and importers to disclose information about the contents of tobacco products to government authorities as well as publicly disclose any toxic constituents and emissions. The NCTD stated that the authority (FMHACA) may take samples of tobacco products at any time for investigative and testing purposes. The authors could not locate any documents or guidelines used by authorities for tobacco product specification. The NCTD also states that ‘modifications or any changes to tobacco product ingredients shall be notified to the authority by the manufacturer or the importer’^12^. However, NCTD did not put any requirements for manufacturers and importers to publicly disclose any toxic constituents and emissions of the tobacco products.

### Packaging and labelling of tobacco products

Article 11 of WHO FCTC, states that packaging and labelling of tobacco products should not promote any misleading and deceptive claims, and prohibits the use of language that creates false impressions that a particular tobacco product is less harmful than another, such as ‘low tar’, ‘light’, and ‘ultra-light’. It also states that each unit packet carries a health warning describing the harmful effects of tobacco use and covers 30–50% of the display area. The NCTD 2015 aligns with the WHO FCTC Article 11 in that it prohibits the use of misleading language such as ‘low tar’, ‘light’, ‘ultra-light’, asks that health warnings be displayed on not less than 30% of each principal display area of the packaging. The NCTD requires that health warnings be presented both in English and Amharic, a national working language.

### Tobacco advertising, promotion and sponsorship

Article 13 of WHO FCTC requires parties to undertake a comprehensive ban on all tobacco advertising, promotion, and sponsorship. The NTCD prohibits all forms of tobacco advertising, promotion, and sponsorship activities, including among other things communication through different platforms such as radio, provision of gifts, supply of free samples and incentive promotions. However, The NCTD does not directly address point-of-sale product display or the sale of tobacco products via the internet. Even though these were indirectly addressed in the document (for example, internet-based tobacco advertising is banned, indicating the prohibition of sale of tobacco products in online platforms), they need to be explicitly banned and indicated in NCTD in order to align with Article 13 of WHO FCTC.

### Sales to and by minors

Article 16 of WHO FCTC requires each party to adopt and implement legislation that prohibits the sale of tobacco products to persons under the age set by the local or national law, prohibits sale of tobacco products on open store shelves and prohibits manufacturing of tobacco products in the form of toys and sweets that appeal to minors. Selling or providing tobacco products, both directly and indirectly, including online sales, to a person under the age of 18 years is prohibited in NCTD 2015 and requires age verification before selling. The manufacture and sale of any tobacco product that resembles toys and sweet products are also prohibited.

### Status of MPOWER implementation in Ethiopia as of 2017

In early 2008, WHO introduced a powerful policy package to assist countries in the implementation of various cost effective interventions and strategies to reduce the demand for tobacco. The policy package, known as MPOWER, entails: 1) Monitoring tobacco use; 2) Protecting the public from tobacco smoke; 3) Offering help to quit smoking; 4) Warn about the dangers of tobacco; 5) Enforcing ban on advertising and promotion; and 6) Raising taxes on tobacco products^17^. There is scarcity of data pertaining to monitoring tobacco use and prevention policies in Ethiopia. A smoking prevalence of 5% was reported in the first comprehensive and nationally representative household survey (Global Adult Tobacco Survey of 2016).

Ethiopia’s current smoke-free legislation suffers from a major policy gap as it allows smoking in designated areas despite that such exceptions are known to dilute strong smoke-free laws. In recent years, some of the major cities of Ethiopia, including Addis Ababa and Mekelle^18,19^, made a significant step towards protecting the public from secondhand smoke exposure by implementing the national smoke-free legislation. However, the overall compliance and implementation of these smoke-free policies at the national level are low and the legislation needs to outlaw designated smoking rooms to achieve a completely smoke-free environment.

Article 14 of the WHO FCTC recommends to institute programs and services to promote smoking cessation and treatment of tobacco dependence. However, access to such services and smoking cessation pharmacotherapies is severely limited in Ethiopia. According to WHO 2017 report, Quitline and other smoking cessation supports are not available in the country. Even though nicotine replacement therapies are listed in the country’s essential drug list, such products are not stocked in most pharmacies.

The use of pictorial health warning labels on tobacco packaging is one of the most effective strategies to raise public awareness about the danger of tobacco use. The NTCD approves about 7 specific health warnings to cover at least 30% of principal display areas of the tobacco product package. The law did not mandate the use of plain packaging and none of the health warnings includes photographs or graphics. Moreover, national mass media anti-tobacco campaigns have not been widely utilised even though such campaigns are recognised as an effective way to raise public awareness and increase quit attempts. The price of cigarettes continues to be affordable in the Ethiopian market, with no price change between 2014 and 2016. The price of Nyala, the most sold brand of cigarettes (standardized to a pack of 20) was less than 1 USD (15 ETB) in 2016 and the total taxes were nearly 19%.

## DISCUSSION

Even though Ethiopia signed the convention to ratify WHO FCTC early in 2004, it took more than a decade (11 years) for the parliament to pass the Bill and issue a comprehensive NTCD, which is not yet fully implemented and/or enforced. A number of structural and policy-level challenges have been identified that delay the ratification and hinder the implementation of WHO FCTC both at regional and country level. These include the government’s economic ties with the tobacco market as the owner of the National Tobacco Enterprise, the sole tobacco producer in the country until 2016, and lack of full political commitment inside the government. WHO FCTC Article 5.3 clearly stated that parties should refrain from involving tobacco industries in the development and implementation of tobacco policies^16^. The Ethiopian government is currently the main stakeholder in the tobacco market via its state-owned tobacco company (SOTC). This, for a long time, has been justified as a source of tax revenue and employment opportunity for the country. This creates a significant and inevitable problem in terms of implementing Article 5.3 of WHO FCTC due to a ‘fundamental and irreconcilable conflict’ between SOTC and public health^20^. This conflict could be further exacerbated if a single state is responsible for overseeing SOTC and implementing tobacco control policies. The guideline for implementation of Article 5.3, which was published in 2008^20^, explicitly asked parties with SOTCs to ‘ensure that the setting and implementing of tobacco control policy are separated from overseeing or managing tobacco industry’ and ‘exclude the regulator from participation in any committees overseeing the implementation of the WHO FCTC or the development of policy and legislation on tobacco control’. In Ethiopia, however, the extent to which these requirements are met is currently unclear. Recently, the government started to partially privatize the tobacco industry. In 2016, JTI became a major stakeholder in Ethiopia’s tobacco market (took 40% of NTE share)^10,21^. The recent growing market interest from giant tobacco industries, such as JTI, poses a significant threat to the implementation of NTCD and other tobacco control policies^21^. The president and CEO of JTI, said: ‘The significant increase in our ownership of NTE shares reaffirms our strong belief in the company and Ethiopia as an increasingly important place to do business in Africa’^10^. Overall, the National level implementation of WHO FCTC is low in Ethiopia^13^ as it is in many African countries^13,22^. In Nigeria, for example, similar policy gaps and legal loopholes such as allowing designated smoking areas in hospitality and transportation venues have been reported^23^.

The NTCD 2015 is Ethiopia’s first comprehensive tobacco control directive, which for most part is WHO FCTC compliant. The legislation prohibits, among other things, sale of flavoured tobacco products including menthol, direct and indirect sale of tobacco products to a person under the age of 18 years and nominally bans all forms of tobacco advertising, promotion, and sponsorship. However, there are major gaps and loopholes in the legislation that the tobacco industry could use to interfere with national tobacco control policies and/or maintain its tobacco market. For instance, the NTCD allows smoking designated rooms and areas in some prohibited public or work places. Allowing smoking designated rooms and areas, despite Article 8 of WHO FCTC, which requires 100% smoke-free places, is being promoted by the tobacco industry for a long time in order to renormalize smoking and make smoking socially acceptable^24^. Most smoking designated areas did not fully adhere to the requirements and standards on how such areas should be organised. This, in turn, led to tobacco smoke pollution even in non-smoking sections of restaurants or other public places with smoking designated areas.

Even though a multi-sectoral National Tobacco Control Coordinating Committee (NTCCC) and national tobacco control strategic plan were established as per Article 5 of WHO FCTC, proposed activities pertaining to the protection of such tobacco control policies from vested interests of tobacco industry (WHO FCTC Article 5.3) are not addressed in NTCD 2015. Implementing WHO FCTC Article 5.3 in countries with SOTCs, such as Ethiopia, is particularly problematic due to the inevitable conflicts of interest^25^. While there are some compelling arguments that privatising SOTCs is the most appropriate policy response to alleviate the inevitable conflicts of interest, the large body of evidence from countries that privatized SOTCs indicate that such policies have resulted in a substantial surge in tobacco consumption due to increased marketing and affordability^26^. Thus, SOTCs may provide unique opportunities for the tobacco end-game as they are more adaptable and responsive according to the political will. In this regard, the government needs to follow a ‘sun-setting’^25^ approach to its tobacco control policies and have a candid discussion with SOTCs regarding mechanisms to end the tobacco epidemic instead of privatising SOTCs, which could ultimately lead to a substantial surge in tobacco consumption.

### The future of tobacco control in Ethiopia

In recent years, encouraging progress is being made by the government and stakeholders to control the surge in tobacco use. The passing of Proclamation No 822/2014 (ratification of WHO FCTC), preparation of the NTCD 2015, and integration of tobacco control strategies into different national health policies (such as the National Drug Master Plan^27^, National Strategic Framework for the Prevention and Control of NCDs^28^ and National Cancer Control Plan^29^), establishment of NTCCC at the federal level and preparation of the first National Tobacco Control Strategic Plan (2017– 2020) are some of the major and historic steps the government took in an effort to see ‘tobacco-free Ethiopians’.

The Ministry of Health, in November 2018, proposed a proclamation called *Food and Medicine Administration Proclamation* that comprises a variety of legislations regarding the sale and use of alcohol, food, narcotic drugs and tobacco products. The new proclamation includes a number of important changes to the 2015 NTCD. Specifically, the following major changes are noted in the proposed proclamation.

- Packaging of tobacco products to contain rotating health warnings and messages that comprise combined images and full-colour to be displayed on no less than 70% of the front and back side of each principal display area of the packaging and labelling.
- Tax levy on tobacco products consistent with the WHO FCTC for the federal government body responsible for initiating the country’s tax policy.
- Tobacco products to be sold only in intact packages containing 20 sticks or consisting of the specified weight, as prescribed by the executive body. This is in contrast to the current legislation that does not ban the sale of a single cigarette stick.
- The proclamation, most importantly, prohibits smoking (use of any kind of tobacco product) ‘in any part of all indoor workplaces, all indoor public places, on all means of public transport, and in all common areas within condominium housings’.

The Bill was deliberated by Council of Ministers on November 2018 and Articles pertaining to tobacco products will come into effect at the twelve month from the date of its adoption. The Bill, if passed by parliament, is a step in the right direction and represents a new era for tobacco control in Ethiopia.

### Tobacco harm reduction as a viable strategy among current smokers

The three-year strategic plan gives emphasis to two main strategic pillars (reducing demand and banning supply of tobacco products), but essentially neglects current smokers and does not include strategies to reduce tobacco related harm for smokers who are unable or unwilling to quit smoking. Tobacco harm reduction offers smokers with the opportunity to reduce tobacco-related harm by completely switching from combustible tobacco cigarettes to a less harmful alternative that delivers the nicotine smokers are addicted to. These alternatives include nicotine replacement therapies (NRTs), low-nitrosamine smokeless tobacco products and electronic nicotine delivery systems (ENDS). Some priority populations who are at high risk of tobacco-related harm such as people with mental health problems, people with substance use disorders and people living with HIV/ AIDS and tuberculosis could benefit from low-risk alternatives to smoking combustible cigarettes, such as electronic cigarettes, as a way to quit smoking or reduce tobacco related harm. Thus, future plans to implement the national strategic plan need to give emphasis to current smokers and provide them with all options to quit smoking including, but not limited to, behavioural support, NRTs, approved smoking cessation medications and low risk alternatives to combustible cigarettes such as ENDS.

As far as the authors are aware, this is the first attempt to document Ethiopia’s tobacco control and prevention efforts pre- and post-ratification of the WHO FCTC. The main methodological limitation that should be taken into account while interpreting the findings is our sole reliance on publicly available and accessible data. Official government websites such as Federal Parliamentary Assembly were not available and/or accessible to retrieve earlier documents regarding Ethiopia’s tobacco legislative history.

## CONCLUSIONS

Being the second most populous African country after Nigeria, Ethiopia represents a huge opportunity for international tobacco companies to invest and recruit new smokers. The NTCD 2015 is Ethiopia’s first comprehensive tobacco control directive, which for most part is WHO FCTC compliant. Major gaps and loopholes in the legislation such as allowing smoking designated rooms and areas in some prohibited public or work places should be addressed in order to halt the tobacco industry from using such loopholes to interfere with national tobacco control policies and/or maintain its tobacco market. The three-year national tobacco control strategic plan should also be institutionalized across various sectors and institutions and ensure that the tobacco industry has no loophole to lobby and weaken the implementation of NCTD and other tobacco control policies. Some of these policy gaps will partially be addressed if the recently proposed *Food and Medicine Administration Proclamation* is passed by parliament. Overall, the government needs to follow a ‘sun-setting’ approach to its tobacco control policies and have a discussion with SOTCs regarding mechanisms to end the tobacco epidemic instead of privatising SOTCs that could ultimately lead to a substantial surge in tobacco consumption.

## CONFLICTS OF INTEREST

Authors have completed and submitted the ICMJE Form for Disclosure of Potential Conflicts of Interest and none was reported.
